# TOM1 family proteins: from cargo sorting to immune dysregulation and cancer

**DOI:** 10.1186/s12964-026-02961-6

**Published:** 2026-05-26

**Authors:** Megan V. Collins, Heljä K. M. Lång, Carla V. Finkielstein, Samppa J. Ryhänen, Elina Ikonen, Daniel G. S. Capelluto

**Affiliations:** 1 Department of Biological Sciences, Virginia Tech, Blacksburg, VA 24061 USA; 2 Protein Signaling Domains Laboratory, Fralin Life Sciences Institute, Virginia Tech, Blacksburg, VA 24061 USA; 3 Children’s Hospital, Pediatric Research Center, University of Helsinki and Helsinki University Hospital, Helsinki, Finland; 4 Fralin Biomedical Research Institute at Virginia Tech Carilion, Roanoke, VA USA; 5 Department of Anatomy and Stem Cells and Metabolism Research Program, Faculty of Medicine, University of Helsinki, Helsinki, Finland; 6 Minerva Foundation Institute for Medical Research, Helsinki, Finland

**Keywords:** TOM1, TOLLIP, Phosphoinositides, ESCRT, Myosin VI, Endosomes, Autophagy, TOL, Immune dysregulation

## Abstract

Intracellular signaling pathways are modulated by ubiquitin-dependent trafficking, in which specific plasma membrane receptors and cytosolic proteins are tagged, internalized, and degraded in the endolysosomal pathway. *T*arget *o*f *M*yb1 (TOM1) family proteins, including TOM1, TOM1-L1, and TOM1-L2 function as early adaptors within the ESCRT-0 machinery to recognize ubiquitinated cargo and coordinate its sorting. TOM1 proteins interact with ubiquitin and accessory proteins, such as TOLLIP, facilitating efficient cargo sequestration and endosomal maturation. These interactions are known to be modulated by pathogen-driven processes, such as *Shigella flexneri*-mediated phosphatidylinositol 5-phosphate accumulation, which can impair TOM1-dependent cargo trafficking. Beyond endosomal sorting, TOM1 contributes to autophagic flux by linking autophagosomes and endosomes through its interaction with the motor protein Myosin VI. Dysregulation of these pathways has been implicated in immune disorders, myocardial ischemia-reperfusion injury, and potentially tumorigenesis. In plants, TOM1-like proteins serve as functional ESCRT-0 analogs, mediating ubiquitin-dependent cargo sorting and integrating stress-responsive signaling. Recent studies have shed light on the modular organization of TOM1, revealing mechanisms of ubiquitin recognition, DXXLL motif function, and complex formation with adaptor proteins. Nonetheless, key questions remain regarding how TOM1 discriminates among ubiquitin linkages, interacts with distinct phosphoinositides under varying physiological conditions, and cooperates with TOLLIP during selective autophagy. Elucidating these mechanisms will advance our understanding of cellular transport and signaling and may reveal novel intervention targets for inflammatory and autoimmune diseases in humans as well as for improving drought tolerance and immune regulation in plants.

## Introduction

 To downregulate signaling pathways, cells employ a tightly controlled process in which membrane-embedded receptors, and likely certain cytosolic proteins [1], are tagged with ubiquitin, internalized *via* endocytosis, and targeted for degradation within the endolysosomal pathway. At the surface of endosomal compartments, the ubiquitin tags on these proteins, collectively referred to as cargo, are recognized by specialized protein assemblies, the *e*ndosomal *s*orting *c*omplexes *r*equired for *t*ransport (ESCRT). The ESCRT machinery drives extensive remodeling of the endosomal membrane, promoting its inward budding and scission to form intralumenal vesicles that sequester cargo for efficient degradation [2]. Among the earliest multifunctional signaling adaptors involved in cargo recognition and sorting are members of the *t*arget *o*f *M*yb1 (TOM1) protein family, which includes TOM1, TOM1-like 1 (TOM1-L1), and TOM1-like 2 (TOM1-L2). This review highlights current knowledge and emerging insights into the roles and regulation of TOM1 family proteins across various physiological and disease-related contexts. In the following sections, we explore how TOM1 mediates ubiquitin-dependent cargo sorting and autophagic flux, and how its function is altered by bacterial infections. We also consider TOM1-related signaling pathways in plants, highlighting conserved and divergent features across kingdoms. Finally, we examine the emerging roles of TOM1 family proteins in immunity and cancer, where dysregulation of cargo trafficking and autophagy contributes to autoimmunity and tumorigenesis.

### TOM1 as a mediator of cargo sorting

#### From ubiquitin recognition to ESCRT-mediated endosomal trafficking

Many cargo proteins are internalized from the cell surface *via* clathrin-coated vesicles and directed to endosomal compartments. Once there, they face two primary fates: recycling to the plasma membrane through the endosomal recycling pathway or degradation *via* the endolysosomal system (in humans and animals) or the vacuole (in plants). These sorting decisions are frequently guided by the addition of ubiquitin to membrane receptors, either as single ubiquitin units (monoubiquitination) or as polyubiquitin chains. Polyubiquitin can form distinct structural types, including compact conformations such as K48-linked chains [3] and more extended, flexible forms like K63-linked chains [4]. A tetraubiquitin chain linked *via* K48 appears to be the shortest chain length necessary for effective targeting to the proteasome [5], whereas a K63-linked hexaubiquitin chain is the minimal length required for recognition by the ESCRT-0 proteins the *h*epatocyte growth factor-*r*egulated tyrosine kinase *s*ubstrate (HRS) and the *s*ignal-*t*ransducing *a*daptor *m*olecules 1/2 (STAM1/2) [6].

Cargo ubiquitination not only marks protein receptors for their eventual fate but also recruits protein sorting machinery. In the degradative pathway, cargo is first recognized by the ESCRT-0 complex, such as HRS-STAM 1/2 or TOM1-*Toll*-*i*nteracting *p*rotein (TOLLIP), which clusters cargo at the surface of the early endosomes [2, 7]. This ESCRT-0 recruitment is primarily driven by the presence of membrane-embedded phosphatidylinositol 3-phosphate (PtdIns3P) [8]. Sequential engagement of the complexes ESCRT-I and ESCRT-II drives invagination of the endosomal membrane, forming intraluminal vesicles that capture the tagged cargo [2]. Deubiquitinases associated with ESCRT components ensure efficient cargo capture but also recycle ubiquitin prior to proteolysis, maintaining cellular ubiquitin homeostasis. Finally, ESCRT-III promotes membrane scission, releasing these vesicles, and the resultant multivesicular bodies (MVBs) fuse with lysosomes (in humans and animals) or vacuoles (in plants), where resident hydrolases degrade cargo [9].

**Fig. 1:**
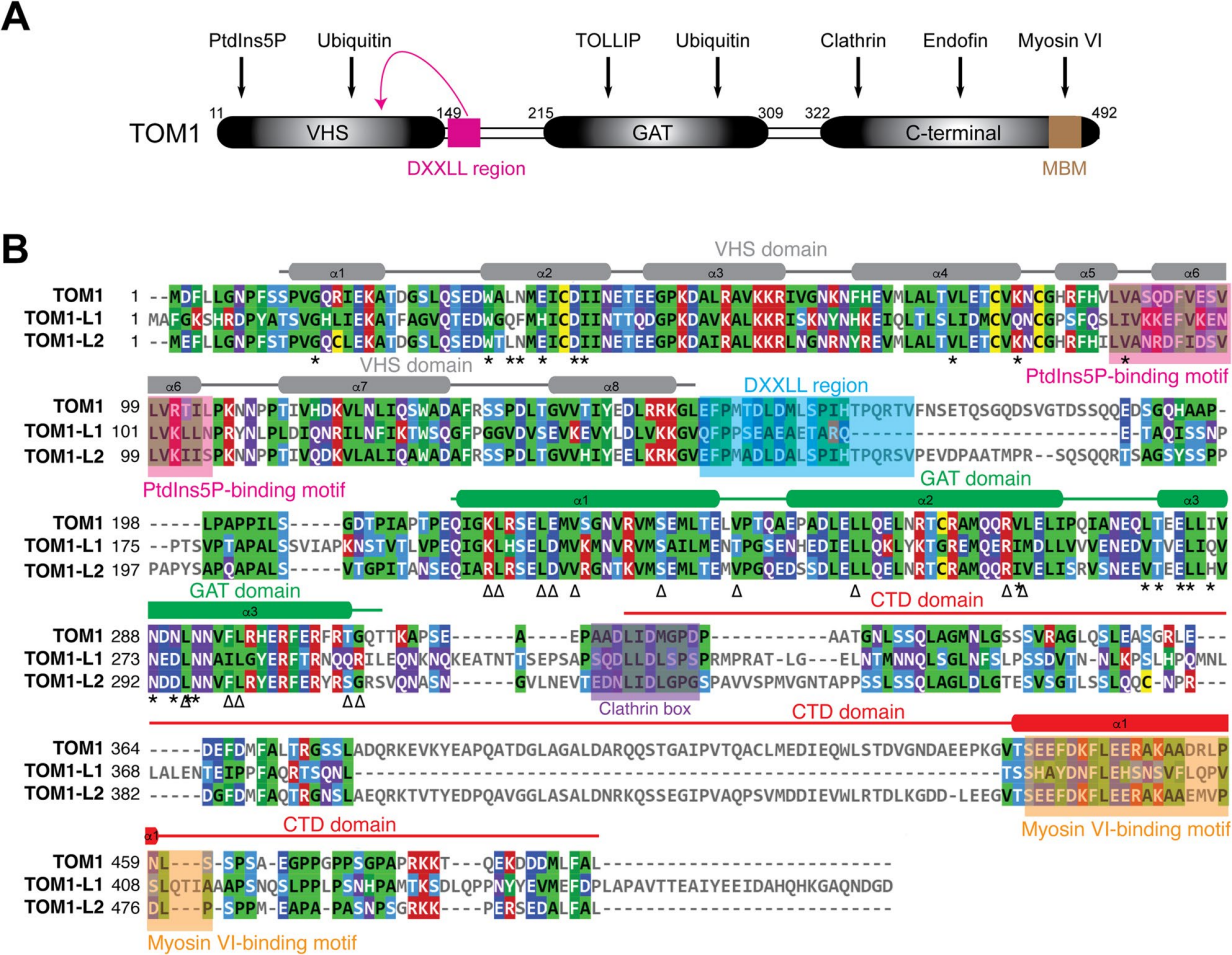
Structural organization, functional regions, and sequence alignment of the TOM1 family proteins. (**A**) Schematic representation of the modular domain organization of TOM1 and its known binding partners. (**B**) Protein sequences of human TOM1 (accession number O60784), TOM1-L1 (accession number O75674), and TOM1-L2 (accession number Q6ZVM7) were aligned using Clustal Omega and visualized using MView. Domain boundaries and secondary structure elements for the VHS, GAT, and CTD domains of TOM1 are indicated in gray, green, and red, respectively. Functional motifs are shaded as follows: PtdIns5P-binding motif in pink, DXXLL region in cyan, clathrin box in purple, and Myosin VI-binding motif in orange. Residues critical for ubiquitin binding are marked with an asterisk, whereas residues involved in TOLLIP binding are indicated by a triangle

In mammals, the canonical ESCRT-0 complex is represented by the HRS and STAM1/2 proteins [10]. These proteins engage cargo through both their ubiquitin-interacting motif (UIM) and *V*ps27, *H*RS, *S*TAM (VHS) domains [11]. Membrane association of the canonical ESCRT-0 complex is mediated by HRS *via* its *F*ab1, *Y*OTB, *V*ac1, *E*EA1 (FYVE) domain, which specifically recognizes PtdIns3P at endosomal membranes [12]. In addition to HRS and STAM1/2, the ubiquitin-binding proteins TOM1, TOM1-L1, and TOM1-L2 are less well-studied components of the ESCRT-0 machinery involved in early cargo transport [13–15]. All members of the TOM1 protein family share a conserved modular organization, consisting of an N-terminal VHS domain, a central *G*G*A*, *T*OM1 (GAT) domain, an acidic-cluster-dileucine (Asp-X-X-Leu-Leu, DXXLL) motif, and a largely disordered C-terminal domain (Fig. 1A). Phylogenetic analyses indicate a close evolutionary relationship among TOM1 orthologs from *Caenorhabditis elegans*, *Drosophila melanogaster*, and *Homo sapiens* (TOM1, TOM1-L1, and TOM1-L2), whereas TOM1-like (TOL) proteins from plants and *G*olgi-localized, *γ*-ear–containing, *A*RF-binding (GGA) proteins show significant divergence [16]. These observations suggest potential functional redundancy among TOM1 family members. In plants, such as *Arabidopsis thaliana* and *Oryza sativa*, the TOL family is notably expanded, with nine genes encoding proteins that contain both VHS and GAT domains [16]. The detailed interactions of TOM1 family proteins with specific binding partners are discussed in the following section.

### TOM1 family proteins as non-canonical ESCRT-0 components

Several lines of evidence collectively support the classification of TOM1 family proteins as bona fide ESCRT-0 components. First, at the structural level, TOM1 family members share the VHS domain characteristic of canonical ESCRT-0 proteins HRS and STAM1/2 [11]. Additionally, the GAT domain of TOM1 proteins provides a second ubiquitin-binding interface, reinforcing their role in the selective capture of ubiquitinated cargo at the endosomal membrane. Second, at the protein interaction level, TOM1 and TOM1-L1 directly interact with the ESCRT-I subunit *t*umor *s*usceptibility *g*ene *101* (TSG101) in both *Dictyostelium* [17] and humans [18], demonstrating physical integration into the ESCRT cascade through a conserved interaction that mirrors the HRS-TSG101 axis of canonical ESCRT-0. Third, functionally, TOM1 family proteins mediate the endosomal sorting of multiple ubiquitinated transmembrane cargoes, including the *i*nter*l*eukin-*1 r*eceptor *1* (IL-1R1) [19], the delta opioid receptor [20], and ciliary G protein-coupled receptors [13] (Table 1), consistent with the cargo-concentration role ascribed to ESCRT-0. Fourth, TOM1 associates with TOLLIP and Endofin (also known as ZFYVE16) (Fig. 1A-B) [21, 22], endosomal proteins that link ubiquitinated cargo to the ESCRT pathway, further placing TOM1 within the ESCRT-0 functional network. Taken together, the structural, interactional, and functional data support TOM1, TOM1-L1, and TOM1-L2 as genuine, albeit non-canonical, members of the ESCRT-0 machinery.

**Table 1:** Overview of the cargoes sorted by TOM1 family proteins, highlighting their diverse molecular partners and the broad range of biological processes they regulate

Cargo	TOM1 family protein partner	Biological function	References
IL-1R1	TOM1	Binds TOLLIP to mediate receptor sorting and delivery to lysosomes	[19]
Mitochondrial-derived vesicles	TOM1	Binds TOLLIP to facilitate trafficking of mitochondrial-derived vesicles to lysosomes	[23]
Delta opioid receptor	TOM1	Required for lysosomal degradation of the receptor	[20]
Fc-gamma-RIIb2	TOM1	Regulates receptor levels and amyloid-β uptake	[24]
GPCRs GPR161, SSTR3, SMO	TOM1-L2	Mediates retrieval of ubiquitinated GPCRs from primary cilia *via* recognition of K63-linked ubiquitin chains	[13]
MT1 MMP	TOM1-L1	Promotes invadopodia formation and invasive behavior	[15]
TAX1BP1	TOM1	Facilitates autophagosome maturation *via* associated cargo	[25]
CALCOCO2/NDP52	TOM1/TOM1-L2	Facilitates autophagosome maturation *via* associated cargo	[26]
OPTN	TOM1/TOM1-L2	Facilitates autophagosome maturation *via* associated cargo	[26]
ABA receptor	Tol proteins	Sorting of the receptor to the vacuoles in plants	[27]
Auxin carrier protein PIN2	Tol proteins	Plant vacuolar sorting and turnover of the transporter	[28]

**Fig. 2:**
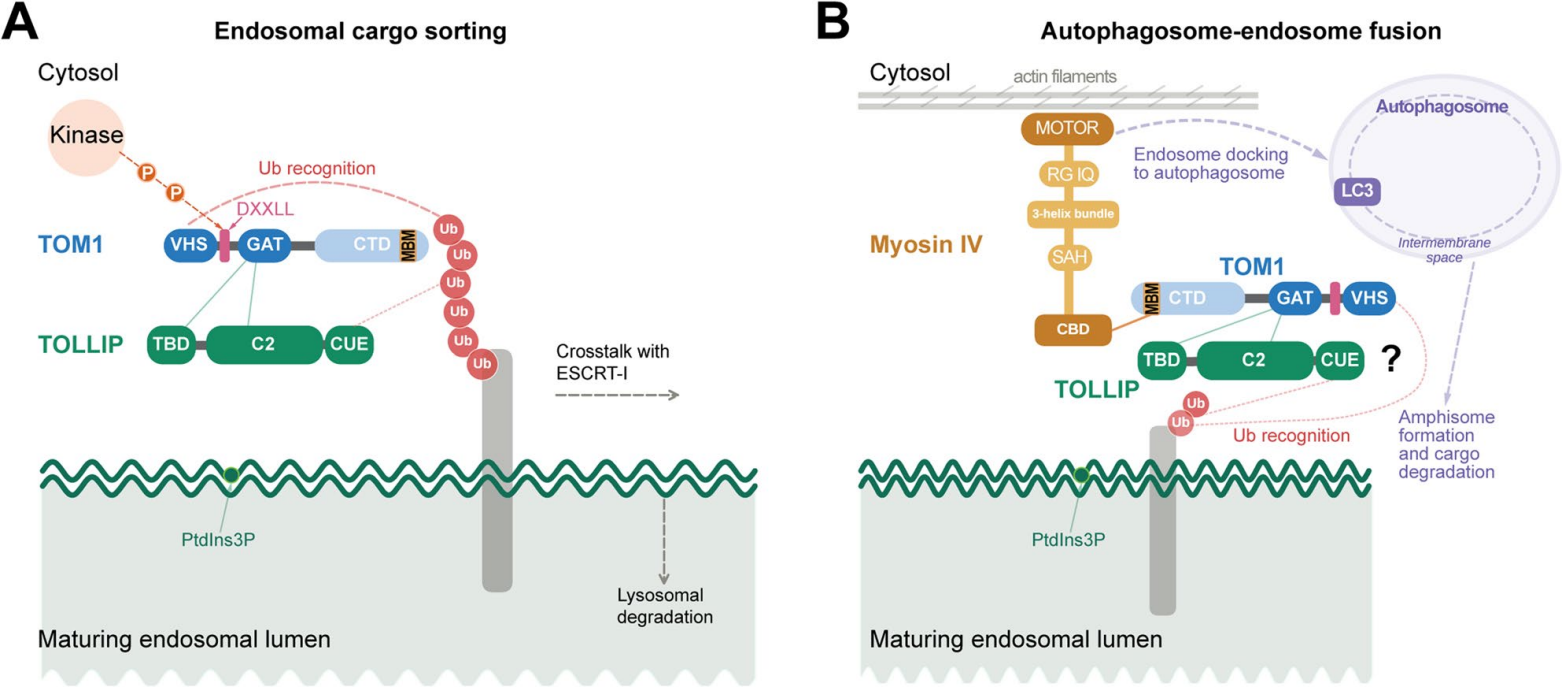
TOM1 functions as a scaffold coordinating ubiquitinated cargo sorting and autophagosome-endosome fusion at the maturing endosome. (**A**) Schematic illustrating the role of TOM1 in endosomal cargo sorting at the maturing endosome. At the cytosolic face of the endosomal membrane, TOLLIP is recruited to endosomal membranes *via* PtdIns3P-binding (not shown). Once recruited, the TOLLIP TBD and C2 domains interact with the TOM1 GAT domain-dependent mechanism. The TOM1-TOLLIP complex engages polyubiquitinated cargo receptors and coordinates crosstalk with ESCRT I components (not shown) to facilitate ubiquitinated cargo delivery for lysosomal degradation. Both TOLLIP CUE and TOM1 VHS domains bind ubiquitin chains, with VHS-mediated binding further enhanced in TOM1 by the DXXLL motif. A kinase-dependent phosphorylation event at the DXXLL region is proposed to downregulate TOM1-ubiquitin interactions. (**B**) Schematic depicting the role of TOM1 in autophagosome-endosome fusion. At the maturing endosome, TOM1 associates with the actin-based motor protein Myosin VI *via* its CTD MBM. Yet to be demonstrated, TOLLIP could be recruited to the PtdIns3P-embedded endosomal membranes to participate in this process by interacting with TOM1 and polyubiquitinated cargo (denoted by a question mark). The TOM1-Myosin VI complex, potentially including TOLLIP, connects the endosome to actin filaments and facilitates its docking to the autophagosome membrane through interaction with LC3. This process drives autophagosome-endosome fusion, leading to amphisome formation and subsequent cargo degradation. It is not known whether other ESCRT components participate in this process, thus making it distinct from the endosomal cargo sorting pathway

TOM1 interacts with both mono- and polyubiquitin through its VHS and GAT domains (Figs. 1 and 2) [29, 30]. Early studies suggested relatively broad specificity, with TOM1 and TOM1-L2 binding K48-, K63-, K29-, and K33-linked polyubiquitin chains [5, 31, 32]. However, accumulating evidence now supports preferential recognition of K63 linkages. Ubiquitin interactome analyses consistently reveal functional associations between TOM1 and K63-ubiquitinated proteins [33], and *in vivo* studies show that TOM1-L2 recognizes G protein-coupled receptors *via* K63-linked modifications in *Chlamydomonas* [13]. The most refined insights come from branch-specific nanobody approaches, which demonstrate that TOM1 and TOM1-L2 selectively recognize K63-linked ubiquitin, either as extended polymers or as di-ubiquitin units within branched chains [34]. Despite this emerging consensus, the molecular mechanisms by which TOM1 discriminates among distinct ubiquitin chain topologies, each likely encoding specific signaling outcomes, remain incompletely understood.

The ubiquitin-binding VHS domain, found in TOM1 and other proteins, belongs to a structurally related superfamily that includes the ENTH and ANTH domains [35]. The canonical VHS domain adopts a right-handed superhelical fold comprising eight α-helices [36, 37], forming a compact bundled structure that facilitates interaction with diverse ligands. Among these helices, α1-α5 and α7 are highly conserved across VHS-containing proteins, suggesting a core scaffolding role, whereas α6 and α8 exhibit greater variability, contributing to ligand specificity [38]. The TOM1 VHS domain retains many structural features of this domain family, although it features a shortened α5 and a distinct orientation of α8 relative to the rest of the domain [14, 36]. The exception is the VHS domain of Tepsin, an accessory protein involved in vesicular trafficking at the *trans*-Golgi network (TGN), lacks α8 altogether and shows no binding to ubiquitin, phosphoinositides, or canonical sorting signals [39].

### Divergent roles of the DXXLL motifs

The DXXLL motif of TOM1, located in the VHS-GAT linker, binds intramolecularly to its own VHS domain, enhancing ubiquitin binding (Fig. 2A) [14]. Although TOM1’s VHS domain structurally resembles those of GGA proteins, it does not recognize DXXLL motifs in *trans* [40], nor does TOM1’s own DXXLL motif bind GGA proteins [41]. To appreciate the significance of this distinction, it is instructive to consider how GGA proteins exploit DXXLL motifs for cargo sorting.

In GGA proteins, interactions with DXXLL motifs present in the cytosolic tails of transmembrane cargo receptors are critical for directing receptor trafficking between the TGN and endosomes [42]. Multiple receptors exploit this mechanism for precise subcellular localization. The low-density lipoprotein receptor-related protein 9 (LRP9) contains two DXXLL motifs in its cytoplasmic tail, both of which are essential for TGN targeting [43, 44]. The presence of dual motifs enables LRP9 to bind GGA proteins with high efficiency, potentially explaining its rapid retrieval from the plasma membrane to the TGN [43]. Similarly, the mannose-6-phosphate receptors, Sortilin-1, sorting-related receptor with A-type repeats, and *β*-site *A*PP *c*leaving *e*nzyme *1* (BACE1) harbor DXXLL motifs that mediate GGA-dependent trafficking between the TGN and endosomes [45–47]. Of note, BACE1 can also be directed to lysosomes *via* ubiquitination, enabling GGA3 recognition and sorting [48].

At the structural level, the α-helices α6 and α8 of GGA VHS domains form a prominent surface groove essential for the recognition of DXXLL motifs present in the cytosolic tails of transmembrane cargo receptors [49, 50]. Structural studies reveal precise molecular requirements: the aspartic acid forms hydrogen bonds with backbone amides in helix 6 and a salt bridge with K130; these interactions are so specific that even substitution with glutamic acid markedly weakens binding [51], whereas the leucine residues engage helices 6 and 8 through hydrophobic contacts, creating a 1:1 binding pocket [49]. TOM1 diverges from this paradigm; instead of mediating cargo recognition, its intramolecular DXXLL-VHS interaction enhances ubiquitin binding (Fig. 2A) [14], contrasting with GGA1 and GGA3, where intramolecular DXXLL-VHS interactions may mediate autoinhibition [52], although this regulatory mechanism remains a matter of debate [53].

Interestingly, phosphorylation adds a dynamic regulatory layer to DXXLL function. TOM1 is phosphorylated at S160 and T164 during cell cycle [54], residues that map within the DXXLL region (Fig. 1B). Phosphomimetic substitutions of TOM1 at these sites result in a threefold decrease in the affinity of TOM1 for ubiquitin [14]. Structural analyses suggest that TOM1 T164 forms transient hydrogen bonds with VHS residues D38 and N80, which become more stable when T164 is phosphorylated [14]. Other adaptor proteins exhibit similar phosphoregulation, albeit with divergent outcomes. Serine phosphorylation upstream of the DXXLL motif enhances the avidity of the cation-independent mannose 6-phosphate receptor for GGA3 [55] and of BACE for GGA1 [56]. At a systems level, adaptor protein-1-associated casein kinase 2 regulates GGA autoinhibition by phosphorylating a serine upstream of the internal DXXLL motif, promoting VHS-hinge interactions that facilitate cargo release [52, 57]. This context-dependent phosphoregulation highlights the versatility of DXXLL-based sorting mechanisms.

### Cargo trafficking partners of TOM1

TOM1 coordinates cargo sorting through partnerships with the endosomal proteins TOLLIP and Endofin, which regulate its cargo recognition and membrane localization. TOLLIP binds TOM1 with high affinity through its N-terminal TOM1-binding domain (TBD) [58], whereas its central C2 domain binds PtdIns3P [59], and its C-terminal *c*oupling of *u*biquitin to *e*ndoplasmic reticulum degradation (CUE) domain [60] mediates TOLLIP dimerization. In addition, both C2 and CUE domains recognize ubiquitin moieties (Fig. 2) [61]. TOM1 associates with TOLLIP *via* a coupled folding and binding mechanism: the first two α-helices of the GAT domain fold TOLLIP TBD, whereas the third α-helix engages the C2 domain and reduces PtdIns3P binding [58]. This competitive displacement may redirect the TOLLIP C2 domain toward ubiquitinated cargo [61]. Given that TOM1 and TOLLIP bind ubiquitin weakly [14, 61, 62], their partnership likely enhances local cargo concentration to facilitate lysosomal targeting. The third GAT α-helix of TOM1 binds ubiquitin without TOLLIP [58, 63], and TOLLIP lacking its TBD still mediates cargo degradation [64].

Endofin is a scaffold protein that localizes to early endosomal membranes by binding PtdIns3P through its central FYVE domain [65] and it recruits TOM1 to these sites through CTD interactions (Fig. 1A-B) [66, 67]. Beyond this interaction, Endofin co-fractionates with canonical ESCRT members, including HRS, TSG101, and the *ub*iquitin-*a*ssociated *p*rotein *1* [68], potentially coordinating sequential ESCRT complex assembly. The Endofin-TOM1 complex also promotes clathrin recruitment to endosomal membranes [67], though the functional significance of this association remains unclear. Together, TOLLIP and Endofin exemplify how TOM1 integrates biochemical and spatial cues to coordinate cargo sorting, while maintaining functional flexibility through independent activities.

### TOM1-mediated control of autophagic flux

Macroautophagy (hereafter autophagy) is a major catabolic pathway that sequesters damaged organelles, protein aggregates, and pathogens in double-membrane autophagosomes for degradation. While mature autophagosomes fuse directly with lysosomes to form degradative autolysosomes, immature autophagosomes often first fuse with late endosomes or MVBs to form amphisomes, which then acquire proteins required for lysosomal fusion [69].

The formation of amphisomes involves multiple proteins, including TOM1, the motor protein Myosin VI, and several ubiquitinated autophagy receptors such as *n*uclear *d*ot *p*rotein *52* kDa (NDP52)/*cal*cium-binding and *co*iled-*co*il domain-containing protein *2* (CALCOCO2), *Tax1*-*b*inding *p*rotein *1* (TAX1BP1), and Optineurin [70] (Table 1; Fig. 2B). TOM1 facilitates this endosome-autophagosome convergence through partnership with Myosin VI, physically bridging these compartments [71]. Myosin VI drives actin-based transport of endosomes toward the minus end [71, 72] but must transition from an autoinhibited monomeric state to an active open conformation before TOM1 can bind [73]. Recent studies have demonstrated that Myosin VI can adopt an active open conformation upon binding to cardiolipin-containing membranes, with Ca^2+^ serving as a stimulatory cofactor in this process [74].

The crystal structure of the C-terminal cargo-binding domain of Myosin VI in complex with TOM1 reveals a unique binding interface distinct from that used for autophagy receptors, suggesting that Myosin VI can bridge autophagosomes and endosomes through these interactions [75]. In this complex, Myosin VI engages TOM1 primarily *via* its WWY motif binding with high affinity to a disordered C-terminal region of TOM1 termed the *M*yosin VI *b*inding *m*otif (MBM; residues 392–463) (Figs. 1A-B and 2B) [75]. Upon interaction, the MBM adopts a continuous α-helix that docks into the solvent-exposed face of the four-stranded β-sheet of Myosin VI, extending toward the α2-helix, with hydrophobic contacts playing a central role in complex stabilization [75]. Notably, most residues within the TOM1 MBM are conserved in TOM1-L1 and TOM1-L2, suggesting a conserved binding mode for Myosin VI across the TOM1 family members.

Dysregulation of the Myosin VI-TOM1 pathway contributes to cardiac pathology through autophagy modulation. During myocardial ischemia-reperfusion injury, Sirtuin 5-mediated desuccinylation of the residue K48 in TOM1 increases its expression, promoting autophagy-associated cell death [76]. Conversely, traditional medicine Astragali radix-Descurainiae semen may offer cardioprotection by reducing Myosin VI and TOM1 expression, thereby limiting excessive autophagy in heart failure models [77]. These findings position the pathway as a therapeutic target where modulation could balance protective versus pathological autophagy in cardiovascular disease.

The functional role of TOLLIP, TOM1’s binding partner, extends to additional autophagy pathways. TOLLIP participates in aggrephagy, the selective clearance of ubiquitinated protein aggregates [78], by binding damaged proteins and facilitating their autophagosomal delivery through *l*ight *c*hain *3* (LC3) interactions and regulation by the lipid kinase VPS38 [64, 79]. Given TOM1’s high-affinity TOLLIP binding and established cargo-sorting functions, its potential involvement in aggrephagy warrants investigation.

### TOM1 function in plant signaling pathways

Plants lack identifiable ESCRT-0 components but have evolved functionally equivalent machinery through the TOM1-like (TOL) protein family [80]. TOL proteins share the VHS-GAT domain architecture characteristic of mammalian TOM1 and, like their animal counterparts, preferentially bind K63-linked ubiquitin chains [7]. Nine *TOL* genes are encoded in the *Arabidopsis* genome [81, 82], with two additional uncharacterized members identified in the algae *Klebsormidium* [7], indicating conservation across plant lineages. The absence of TOLLIP orthologs in plants suggest that TOL proteins have adopted alternative regulatory mechanisms. Consistent with this, a subset of TOLs, TOL2, TOL3, TOL6, and TOL9, functionally associates with the TPLATE complex, a membrane trafficking machinery that mediates plasma membrane cargo internalization [83]. Direct interaction between the TPLATE component *A*. *thaliana* *e*poxide *h*ydrolase *1* (AtEH1) and TOL6/TOL9 promotes condensate formation that drives clathrin-mediated endocytosis [84] linking TOL proteins to early stages of cargo uptake. The subcellular distribution of TOL proteins further supports functional specialization: TOL3, TOL6, and TOL9 localize to the plasma membrane, whereas remaining TOL members are cytoplasmic [28, 85], reflecting the spatial organization of their respective cargo pools.

Functional studies confirm that TOL proteins serve as essential cargo sorting adaptors in plants. Simultaneous disruption of five *TOL* genes (*TOL2*, *TOL3*, *TOL5*, *TOL6*, and *TOL9*) results in developmental defects and failure to degrade plasma membrane proteins [28, 86], indicating non-redundant roles among family members. Interaction with the ESCRT-I component *v*acuolar *p*rotein-*s*orting-associated protein *23 A* (VPS23A), demonstrated for TOL2 and TOL6 [85], positions these proteins as direct links between cargo recognition and downstream ESCRT-mediated membrane remodeling. At the molecular level, the mechanism of ubiquitin recognition appears to be evolutionary conserved. Tryptophan 25 in the TOL6 VHS domain ubiquitin-binding site [85] corresponds to W30 in the VHS domain of TOM1 [14], a residue known to be critical for TOM1’s interaction with ubiquitin.

Beyond cargo sorting, TOL proteins appear to play a role in early plant responses to drought stress, as their expression is significantly upregulated under such conditions [87]. Abscisic acid (ABA) is the primary hormonal regulator of plant adaptive responses to water deficit, mediating physiological and transcriptional adjustments that enhance survival. ABA receptors are selectively trafficked to the vacuole for degradation *via* the plant ESCRT machinery [88, 89] and TOL proteins appear central to this process (Table 1). Expression of *TOL* genes is significantly upregulated under drought conditions [27], consistent with increased demand for ABA receptor turnover. The functional importance of this regulation is demonstrated by *tol2/3/5/6* quadruple mutant plants [27], indicating that these TOL members normally constrain ABA signaling by promoting receptor degradation. Collectively, these findings establish TOL proteins to function as multifunctional adaptors that integrate ubiquitin recognition, membrane trafficking, and stress-responsive signaling, reinforcing their evolutionary role as plant-specific substitutes for canonical ESCRT-0 components.

Taken together, the plant TOL system offers several conceptual insights into mammalian TOM1 biology. The conservation of the VHS domain tryptophan residue critical for ubiquitin binding across kingdoms indicates that this contact surface is under strong selective pressure, validating its functional importance in mammalian TOM1. Conversely, the divergence in regulatory partners, namely TOL proteins associating with the TPLATE complex rather than TOLLIP, illustrates how the same core cargo-sorting logic can be rewired to accommodate lineage-specific membrane trafficking demands, suggesting that mammalian TOM1’s partnership with TOLLIP represents one solution among several to the problem of coupling ubiquitin recognition to ESCRT recruitment.

### Pathogen-driven modulation of TOM1 function

*Shigella flexneri* subverts host trafficking by remodeling endosomal phosphoinositide composition. Upon invasion, the bacterium secretes the lipid phosphatase *i*nvasion *p*lasmid *g*ene *D* (IpgD), which converts plasma membrane PtdIns(4,5)P₂ into PtdIns5P [90]. This lipid shift has cascading consequences in the host cell. Rab11a is recruited to the bacteria-containing vacuole, facilitating bacterial release [91] and ligand-independent *e*pidermal *g*rowth *f*actor *r*eceptor (EGFR) internalization and endosomal targeting are promoted, sustaining cell survival signaling [90] and TOM1 is sequestered at PtdIns5P-enriched endosomes, impairing EGFR degradation and fluid-phase endocytosis [92]. Although the TOM1 family member TOM1-L1 is known to mediate the internalization of activated EGFR under physiological conditions [93, 94], TOM1 itself has not been shown to play such a role. Rather, the sequestration of TOM1 at PtdIns5P-enriched endosomes during *S. flexneri* infection likely traps TOM1, preventing its engagement with the canonical ESCRT machinery required for EGFR sorting into intraluminal vesicles, thereby impairing rather than promoting degradative trafficking. Overall, these events disrupt normal receptor turnover while simultaneously activating pro-survival pathways, thus, creating conditions that favor bacterial persistence.

Under physiological conditions, the TOM1 family member TOM1-L1 controls EGFR-dependent cell-survival pathways by operating within a subset of clathrin-coated pits that serve as signaling platforms to drive phosphatidylinositol 3,4-bisphosphate [PtdIns(3,4)P₂]-dependent Akt2 activation [94]. TOM1-L1 functions as an adaptor that is pre-associated with longer-lived clathrin-coated pits through direct clathrin binding and, upon EGF stimulation, helps recruit the Src-family kinase Fyn to those pits. The TOM1-L1-Fyn complex recruits the lipid phosphatase *S*rc *h*omology 2 domain-containing *i*nositol 5-*p*hosphatase 2 (SHIP*2*) to generate PtdIns(3,4)P₂, enabling specific activation of Akt2 [94]. Because SHIP2 converts phosphatidylinositol (3,4,5)-trisphosphate into PtdIns(3,4)P₂ [95], it, unlike the bacterial phosphatase IpgD, lowers the amount of lipid available for conversion to PtdIns5P during cell-survival signaling.

**Fig. 3:**
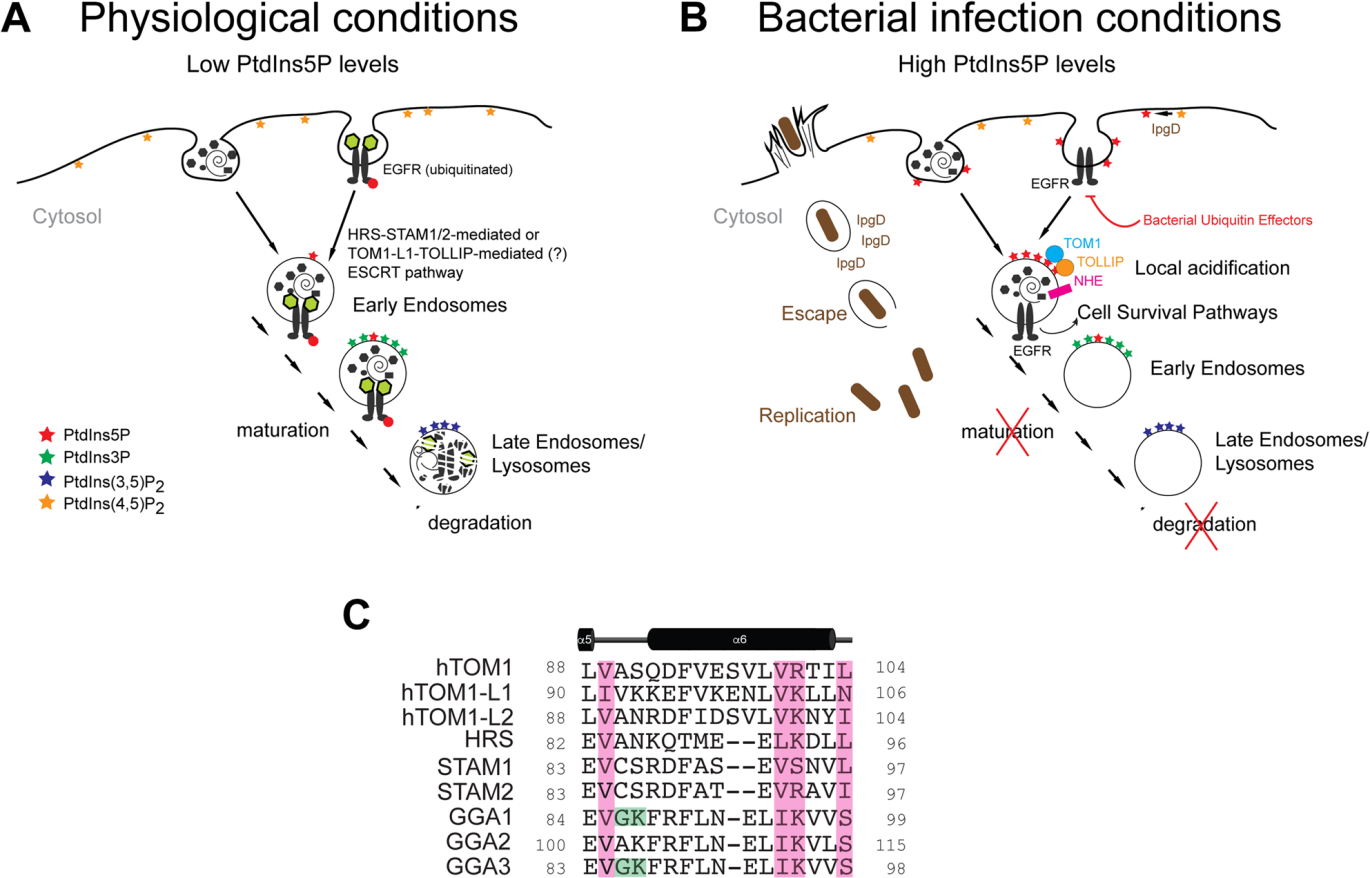
The role of mammalian TOM1 family proteins under physiological and pathological conditions. (**A**) Under physiological conditions, EGFR signaling is attenuated through ubiquitination, followed by clathrin-mediated internalization and trafficking to early endosomes. From there, PtdIns3P-dependent ESCRT proteins direct EGFR toward lysosomal degradation. Whereas TOM1-L1 has been shown to facilitate EGFR transport, the role of TOLLIP is this process remains unknown. The subcellular distribution of other phosphoinositides is also indicated. (**B**) During infection, *S. flexneri* enters epithelial cells *via* macropinocytosis and secretes the lipid phosphatase IpgD, which converts plasma membrane PtdIns(4,5)P₂ into PtdIns5P. The accumulation of PtdIns5P at the plasma membrane enhances the endocytosis of ligand-free EGFR, whose vesicles subsequently fuse with early endosomes. Elevated PtdIns5P levels may stimulate NHE pump activity, inducing local cytosolic acidification that promotes TOM1 recruitment to PtdIns5P-enriched membrane sites, potentially in cooperation with TOLLIP. The resulting TOM1-TOLLIP complex may then activate ligand-independent EGFR-dependent cell survival pathways. Concurrently, endosomal maturation is impaired, decreasing protein turnover at lysosomes. Collectively, these alterations create a cellular environment that supports bacterial survival and replication. (**C**) Sequence alignment of VHS-containing proteins implicated in cargo trafficking. Protein accession numbers are as follows: HRS (O14964), STAM1 (Q92783), STAM2 (O75886), GGA1 (Q9UJY5), GGA2 (Q9UJY4), and GGA3 (Q9NZ52). Alignment was taken from [105]. The four residues identified as critical for PtdIns5P binding in the TOM1 VHS domain are highlighted in pink, whereas the conserved Gly-Lys sequence characteristic of the Walker A motif is shown in green

Phosphoinositides play a central role in organelle function, as the activity of V-ATPases and *N*a⁺/*H*⁺ *e*xchangers (NHEs) depend on their interaction with specific phosphoinositide species. Endosomal maturation is tightly coupled to lumenal acidification, driven largely by V-type H^+^-ATPase activity, with NHEs providing an additional layer of regulation by exchanging lumenal protons for cytosolic Na^+^ [96]. Acute depletion of the phosphoinositide phosphatase Sac1 disrupts TGN phosphoinositide homeostasis, leading to V-ATPase disassembly, organellar deacidification, and impaired Golgi integrity [97]. NHE activity is similarly sensitive to phosphoinositide composition. Phosphatidylinositol 3,5-bisphosphate promotes dimerization of *Equus caballus* NHE9, enhancing its ion transport activity in endosomes [98], and in yeast, the endosomal NHE Nhx1 is required for recruitment of the phosphoinositide-binding ESCRT-0 protein Vps27p to endosomal membranes [99]. NHE dysfunction itself disrupts endosomal maturation, as NHE8 depletion impairs MVB cargo sorting [100], and NHE6 loss causes endosomal overacidification and impaired maturation [101, 102]. Interestingly, among VHS- and GAT-containing proteins, GGA1 retains NHE6 at endosomal membranes through direct cytosolic tail interaction [103], raising the possibility that TOM1 may engage in a comparable, yet uncharacterized, association with NHEs to regulate endosomal pH and maturation. This hypothesis is supported by the observation that TOM1 preferentially binds PtdIns5P-containing liposomes under acidic conditions, with its VHS domain primarily driving this interaction [14], suggesting that local lipid composition and pH dynamics control TOM1-dependent cargo recognition and trafficking. A comparable pH-sensitive mechanism is observed for HRS, a component of the canonical ESCRT-0 complex, which preferentially associates with endosomal membranes under acidic conditions [104]. Taken together, these observations support a model in which *S. flexneri* generates a suitable endosomal environment, enriched in PtdIns5P and exhibiting altered pH, that sequesters TOM1 and promotes ligand-independent EGFR signaling to sustain host cell survival (Fig. 3A-B).

The PtdIns5P-binding site within the TOM1 VHS domain has been mapped to the region spanning α-helices 5 and 6, where residues V89, V100, R101, and L104 primarily mediate lipid binding [14]. This site corresponds to a conserved region identified in other PtdIns5P-binding proteins [106], although TOM1 lacks their characteristic Walker A GK motif [14]. Of note, three of those four TOM1 VHS residues are conserved across VHS-containing proteins, including HRS, STAM1/2, and GGA1/2/3 and that GGA1 and GGA3 harbor the GK sequence (Fig. 3C). Together, these observations raise the possibility that PtdIns5P recognition is a general property of VHS domains, potentially expanding their potential functional repertoire beyond phosphoinositide-mediated trafficking pathways.

TOM1 family proteins participate in host defense against additional pathogens through distinct mechanisms. During *Staphylococcus aureus* infection, complement anaphylatoxin C5a impairs neutrophil phosphoprotein signaling, reducing TOM1 and Endofin phosphorylation alongside VPS34 activity, leading to a depletion of PtdIns3P [107] and, likely, disrupted phagosomal maturation. Separately, TOM1-L2 contributes to xenophagy by facilitating Rab41-mediated ESCRT recruitment *via* its VHS and GAT domains, promoting membrane repair following bacterial toxin damage [108]. Specifically, TOM1-L2, *via* its VHS and GAT domains, acts as a key adaptor linking Rab41 to ESCRT components, promoting efficient membrane repair upon bacterial toxin-induced damage.

In plants, TOL proteins fulfill analogous immune functions. Silencing a *TOL* gene in *Nicotiana benthamiana* reduces bacterial pathogen growth [109], while the fungus *Phytophthora infestans* exploits this system through its effector protein AVRcap1b, which directly binds Tol9a to suppress *N*LR *r*equired for *c*ell death *2* (NRC2) and NRC3 immune signaling and the hypersensitive response [110]. Together, these examples illustrate how TOM1 family proteins across kingdoms function at the interface of trafficking and immunity, and how pathogens have evolved strategies to subvert these functions.

### Significance of TOM1 in immune dysregulation

The immune system is also regulated by the organization and activities of intracellular endosomal compartments associated with cargo sorting, membrane trafficking, and signaling. This enables the generation of sufficient immune responses to fight pathogens while also preventing inappropriate responses which cause autoimmunity or excessive inflammation [111]. TOM1 has been shown to suppress important inflammatory signaling pathways induced by IL-1β and tumor necrosis factor-α via its VHS domain *in vitro* [112]. *In vivo* experiments show that TOM1-L2 hypomorphic mice are prone to infections and tumors, as well as abnormal immunologic responses [113], supporting the role of other members of this protein family in controlling immune responses (Table 2). Together, TOLLIP and TOM1 have been shown to sort IL-1R1 at late endosomes for degradation, which has been proposed to play a role in regulating IL-1β-triggered inflammation [19]. This regulatory axis is particularly relevant in the context of neurodegeneration, where IL-1β signaling has emerged as a key contributor to disease pathology. Alzheimer’s disease (AD) is increasingly recognized as having a prominent neuroinflammatory component, with chronic activation of microglia and astrocytes, dysregulated cytokine signaling, particularly involving IL-1β, and persistent immune dysregulation now considered central to its pathogenesis rather than merely secondary consequences of amyloid accumulation [114]. A study investigating the mechanisms of AD revealed decreased levels of TOM1, but not TOLLIP, in brains of AD patients, accompanied by an increase in IL-1R1 and IL-1β levels (Table 2) [115]. As a negative regulator of IL-1R1 signaling [19], reduced TOM1 expression would be expected to sustain and amplify IL-1β-driven inflammatory cascades that contribute to microglial dysfunction and neuronal damage in the disease. The impairment of TOM1-mediated resolution of IL-1β signaling thus represents a plausible mechanism through which immune dysregulation is maintained in AD brain tissue.

**Table 2:** Overview of dysfunctions associated with TOM1 family proteins

Protein	Disease / condition	Category		Dysfunction	References
TOM1	Alzheimer’s disease (AD)	Neuro inflammation		Reduced TOM1 expression in hippocampi of AD patients; elevated IL-1R1 and IL-1β levels; impaired IL-1R1 lysosomal degradation; enhanced Aβ accumulation; cognitive decline.	[24, 115]
TOM1	Childhood-onset multi-organ autoimmunity and combined immunodeficiency	Autoimmunity		Missense variant p.G307D in the GAT domain disrupts TOM1-TOLLIP cellular co-localization; impaired TOLLIP PtdIns3P regulation; defective autophagosome-lysosome fusion; excessive inflammatory pathway activation.	[116, 117]
TOM1	Immune dysregulation, polyendocrinopathy and enteropathy	Autoimmunity		Heterozygous deletion of VHS domain residues 100–122; profound immune dysregulation.	[118]
TOM1	*Shigella flexneri* infection	Infection		TOM1 sequestered at IpgD-generated PtdIns5P-enriched endosomes; impairs EGFR degradation and fluid-phase endocytosis; prevents ESCRT-mediated EGFR sorting into intraluminal vesicles; promotes bacterial persistence.	[92]
TOM1	*Staphylococcus aureus* infection	Infection		Complement C5a impairs neutrophil phosphoprotein signaling, reducing TOM1 and Endofin phosphorylation	[107]
TOM1	Renal clear cell carcinoma	Cancer		Altered transcript-level expression is prognostic	www.proteinatlas.org
TOM1-L1	HER2+/ER+ breast cancer with early metastatic relapse	Cancer		TOM1-L1 co-amplified with ERBB2; overexpression enhances invasiveness *via* MT1-MMP-dependent invadopodia activation; promotes extracellular matrix degradation and metastatic progression.	[15]
TOM1-L1	Glioblastoma multiforme	Cancer		Prognostic at transcript level; functional silencing impairs cell proliferation, colony formation, migration and invasion *in vitro*; overexpression facilitates malignant progression *via* PTM pathway dysregulation.	[119]
TOM1-L1	Esophageal squamous carcinoma	Cancer		Ectopic overexpression promotes tumor growth, colony formation, migration and invasion *in vitro*.	[120]
TOM1-L1	Renal clear cell carcinoma	Cancer		Altered transcript-level expression is prognostic	www.proteinatlas.org
TOM1-L2	Bacterial xenophagy / membrane damage	Infection		TOM1-L2 acts as adaptor linking Rab41 to ESCRT machinery; promotes VPS4 recruitment; required for xenophagolysosome acidification maintenance and membrane repair after bacterial cytolysin damage.	[108]
TOM1-L2	Immune deficiency and tumor susceptibility		Immunity	TOM1-L2 hypomorphic mice are prone to infections, tumors, and abnormal immunologic responses.	[113]
TOM1-L2	Renal clear cell carcinoma		Cancer	Altered transcript-level expression is prognostic.	www.proteinatlas.org

Human genetic studies have definitively established TOM1 as essential for immune homeostasis and revealed mechanistic insights into its function. A missense mutation (p.G307D) in the GAT domain causes severe childhood-onset multi-organ autoimmunity and combined immunodeficiency (Table 2) [116]. Patient-derived cells harboring this variant exhibit a disrupted TOM-TOLLIP interaction, impaired regulation of TOLLIP’s PtdIns3P binding, and defective autophagosome-lysosome fusion [116, 117], demonstrating that GAT domain integrity is required for multiple TOM1 functions.

A second variant, a heterozygous deletion of VHS domain encompassing residues 100–122, produces profound immune dysregulation, polyendocrinopathy, and enteropathy in an unrelated pediatric patient (Table 2) [118]. Structurally, this deletion spans α-helix 6 through α-helices 7 and 8, which stabilize the VHS fold [22], and is predicted to destabilize the hydrophobic core while disrupting both the DXXLL motif and ubiquitin recognition [14]. These complementary loss-of-function mutations establish that both TOM1’s cargo recognition (VHS and GAT) and adaptor engagement (GAT) functions are non-redundant and essential for preventing human immune dysregulation.

### The emerging role of TOM1 in tumor biology

TOM1 family proteins contribute to cancer progression by redirecting their trafficking functions toward invasive behaviors. TOM1-L1 is co-amplified at the genomic level with the *er*ythro*b*lastic oncogene *B2* (*ERBB2) (HER2)* oncogene in a subgroup of human HER2+/Estrogen receptor (ER)+ breast cancers characterized by early metastatic relapse [15]; this co-amplification is associated with transcriptional upregulation of TOM1-L1, and the combined genomic and expression data suggest that TOM1-L1 overexpression functionally contributes to the metastatic phenotype of this subgroup. The pro-tumoral activity of TOM1-L1 involves membrane type-1 *m*atrix *m*etallo*p*rotease (MMP)-dependent activation of invadopodia, e.g., membrane protrusions engaged in extracellular matrix degradation (Table 1) [15]. Mechanistically, TOM1-L1 pro-invasive activity requires ERBB2-induced interaction of its GAT domain with TOLLIP [121]. As described for TOM1 [122], TOLLIP regulates TOM1-L1 docking to the *Ra*s-related protein Ra*b*-*7*a and MMP-containing endosomes, promoting MMP trafficking to invadopodia [15]. Thus, TOM1-L1 could function similarly as TOM1, which associates with the Myosin VI motor protein, delivering endosomes to autophagosomes and facilitating autophagosome maturation [26].

The oncogenic potential of TOM1 family proteins extends beyond breast cancer (www.proteinatlas.org). All three family members, TOM1, TOM1-L1 and TOM1-L2, are prognostic markers at the transcript levels in renal clear cell carcinoma, and TOM1-L1 is also prognostic at the transcript level in glioblastoma multiforme based on patient cohort analyses. Interestingly, in recent reports functional silencing of TOM1-L1 in glioblastoma multiforme cell lines impaired cell proliferation, colony formation, migration and invasion *in vitro* [119], while ectopic TOM1-L1 overexpression promoted the growth, colony formation, migration and invasion of esophageal squamous carcinoma cells [120]. A summary of the association between TOM1 family proteins and cancer is provided in Table 2. Together, these findings suggest that TOM1 family proteins may play broader roles in malignant progression of than so far anticipated.

## Conclusions and future perspectives

TOM1 and its family members have emerged as protein adaptors at the crossroads of ubiquitin-dependent trafficking, autophagy, and immune regulation. Initially recognized as components of the ESCRT-0 machinery, TOM1 proteins are now appreciated as multifunctional players that integrate signals from ubiquitin, phosphoinositides, and organelle-based adaptor proteins to coordinate cargo transport and degradation. Their roles extend from endosomal sorting and autophagosome maturation to specialized processes such as bacterial xenophagy in animals and stress responses in plants. This versatility reflects the modular evolutionary conserved nature of TOM1, which enables interaction with partners including TOLLIP, Endofin, Myosin VI, and, under bacterial infection conditions, phosphatidylinositol 5-phosphate. Dysregulation of these interactions, whether through pathogen interference, post-translational modifications, or genetic mutations, has consequences ranging from reduced receptor turnover to immune dysfunction. In addition, the identification of a DXXLL motif in TOM1 [14], potentially enhancing cargo recognition, opens new avenues for investigating how VHS domains from other proteins recognize TOM1 DXXLL motifs and, conversely, how TOM1 VHS domains may recognize DXXLL sequences in *trans*.

Despite major advances in understanding TOM1 protein functions, many key questions remain unresolved. The structural basis by which TOM1 discriminates among ubiquitin chain topologies is still poorly understood, as is the full extent of its ability to recognize distinct phosphoinositides under variable pH conditions or by forming complexes with other proteins. Given their high-affinity interaction, the potential cooperative role of TOM1 and TOLLIP in selective autophagy also warrants further investigation. In particular, it remains to be determined whether TOLLIP, which participates in autophagy, contributes to Myosin VI-mediated bridging between endosomes and autophagosomes. Although TOM1 and TOLLIP can work independently, a major open question is how the TOM1-TOLLIP complex dissociates to allow the proteins to function separately. Moreover, pathogen-driven subversion of TOM1 function underscores the need to better understand how lipid environments and ion fluxes regulate TOM1 membrane recruitment and activity. Across kingdoms, the evolutionary expansion of plant TOL proteins as ESCRT-0 substitutes raises parallel questions about their integration of trafficking with hormonal stress signaling.

Marked progress has been made with the structural resolution of the interaction motifs that mediate the formation of the TOM1-Myosin VI complex. Still, future studies should aim to resolve high-resolution structures of the TOM1 family proteins in complex with ubiquitin chains and adaptor proteins to define the conformational states that drive their diverse functions. From a translational perspective, TOM1 represents a compelling therapeutic target. Its modular architecture offers multiple intervention points; small molecules or peptide mimetics targeting the VHS, GAT, or DXXLL motif could disrupt cargo recognition, whereas stabilizing TOM1-TOLLIP interactions may restore autophagic flux in inflammatory or infectious contexts. However, achieving selectivity among TOM1 family members and defining the precise disease contexts in which their activity is dysregulated represent key challenges. By bridging intracellular trafficking and disease-associated pathways, TOM1 family proteins remain a rich source of biological and clinical insight.

## Data Availability

No datasets were generated or analyzed during the current study.

## Abbreviations

CUE: Coupling of ubiquitin to endoplasmic reticulum degradation
DXXLL: Asp-X-X-Leu-Leu
EGFR: Epidermal growth factor receptor
ESCRT: Endosomal Sorting Complexes Required for Transport
FYVE: Fab1, YOTB, Vac1, EEA1
GAT: GGA, TOM1
GGA1/2/3: Golgi-localized, γ-adaptin ear containing, ARF-binding protein 1/2/3
HRS: Hepatocyte growth factor-regulated tyrosine kinase substrate
MBM: Myosin VI-binding motif
MVB: Multivesicular bodies
NHE: Na^+^/H^+^ exchanger
PtdIns(3,5)P₂: Phosphatidylinositol 3,5-bisphosphate
PtdIns(4,5)P₂: Phosphatidylinositol 4,5-bisphosphate
PtdIns3P: Phosphatidylinositol 3-phosphate
PtdIns5P: Phosphatidylinositol 5-phosphate
STAM1/2: Signal transducing adapter molecule 1/2
TBD: TOM1-binding domain
TGN: Trans-Golgi network
TOL: TOM1-like protein
TOLLIP: Toll-interacting protein
TOM1: Target of Myb protein 1
TOM1-L1: Target of Myb protein 1-like 1
TOM1-L2: Target of Myb protein 1-like 2
VHS: Vps27, Hrs, STAM domain
